# *Escherichia coli* with a Tunable Point Mutation Rate for Evolution Experiments

**DOI:** 10.1534/g3.120.401124

**Published:** 2020-06-05

**Authors:** Nicholas A. Sherer, Thomas E. Kuhlman

**Affiliations:** * Department of Physics and; ^†^ Center for the Physics of Living Cells, University of Illinois at Urbana-Champaign, Urbana, IL 61801, and; ^‡^ Department of Physics and Astronomy, University of California Riverside, Riverside, CA 92521

**Keywords:** Experimental Evolution, Mismatch Repair

## Abstract

The mutation rate and mutations’ effects on fitness are crucial to evolution. Mutation rates are under selection due to linkage between mutation rate modifiers and mutations’ effects on fitness. The linkage between a higher mutation rate and more beneficial mutations selects for higher mutation rates, while the linkage between a higher mutation rate and more deleterious mutations selects for lower mutation rates. The net direction of selection on mutations rates depends on the fitness landscape, and a great deal of work has elucidated the fitness landscapes of mutations. However, tests of the effect of varying a mutation rate on evolution in a single organism in a single environment have been difficult. This has been studied using strains of antimutators and mutators, but these strains may differ in additional ways and typically do not allow for continuous variation of the mutation rate. To help investigate the effects of the mutation rate on evolution, we have genetically engineered a strain of *Escherichia coli* with a point mutation rate that can be smoothly varied over two orders of magnitude. We did this by engineering a strain with inducible control of the mismatch repair proteins MutH and MutL. We used this strain in an approximately 350 generation evolution experiment with controlled variation of the mutation rate. We confirmed the construct and the mutation rate were stable over this time. Sequencing evolved strains revealed a higher number of single nucleotide polymorphisms at higher mutations rates, likely due to either the beneficial effects of these mutations or their linkage to beneficial mutations.

Mutation rates are critical to determining the course of evolution, and mismatch repair systems are important systems affecting mutation rates by correcting errors in DNA replication before they become mutations. Furthermore, the mutation rate acts as a parameter in evolutionary models describing regimes from classical hard sweeps where alleles in a population fix one by one to opposite evolutionary regimes, where selection proceeds from standing genetic variation, or in a traveling wave, where clonal interference occurs or lineages with multiple mutations are competing at the same time (Desai and Fisher 2007). The mutation rate is therefore fundamental in determining the the amount of standing genetic variation in a population (Charlesworth 2015; Lynch *et al.* 1998; Huang *et al.* 2016). The combination of the mutation rate and population size also may move a population between different evolutionary regimes; large populations such as laboratory cultures of bacteria or yeast with sufficiently high mutation rates often evolve in a regime of clonal interference (Campos and de Oliveira 2004; Bollback and Huelsenbeck 2007; Fogle *et al.* 2008; Lang *et al.* 2013).

The ubiquitous importance of the mutation rate to natural selection means the mutation rate itself is under selective pressure. Both eukaryotes and bacteria have mismatch repair systems which reduce the mutation rate below what it would be in the absence of mismatch repair (Lin *et al.* 2007; Li 2008; Fukui 2010). Mismatch repair systems fix mistakes in DNA replication where an incorrect nucleotide on a daughter strand is paired with the nucleotide on the template strand. Two proteins used in mismatch repair in *E. coli*, MutS and MutL, have homologs in most bacteria and eukaryotes. MutS dimers recognize mismatches and bind them, while MutL dimers bind MutS dimers. In most species, MutL homologs also have endonuclease activity that helps remove the DNA strand with the mismatch. However, in *E. coli* the endonuclease MutH is recruited by a complex of MutS and MutL and nicks the newly synthesized strand of DNA with the mismatch. *E. coli* with one of these three mismatch repair genes knocked out have a mutator phenotype with mutation rates elevated by two orders of magnitude compared to *E. coli* with mismatch repair (Marinus (2010); Elez *et al.* (2012)).

Mismatch repair deficient strains have been important in experiments investigating the effects of mutation rates on evolution and have evolved in evolution experiments. MutS knockout strains deficient in mismatch repair were used along with wildtype in evolution experiments to investigate the effect of the mutation rate on *E. coli* colonization and adaptation in mouse guts by (Giraud *et al.* 2001). (Sniegowski *et al.* 1997) found that 3 of 12 replicate lines evolved in the Lenski *E. coli* long term evolution experiment had become mutators due to changes related to mismatch repair over the course of their evolution. After many more generations of the long term evolution experiment, there was a 4^th^ mismatch repair mutator as well as 2 other types of hypermutators (Tenaillon *et al.* 2016). By engineering four *E. coli* strains to have unique fixed mutations with a mixture of mismatch repair and polymerase proofreading mutants, (Sprouffske *et al.* 2018) recently investigated the effects of the mutation rate on adaptation. They found that, while populations with higher mutation rates accumulated greater genetic diversity, the diversity conveyed benefits only for modestly increased mutations rates. Strains with the highest mutation rates showed reduced adaptation and experienced a decrease in their mutation rate.

The evolution of mismatch defective strains in past evolution experiments and their successful use in probing evolutionary dynamics suggested we could make a useful strain for evolution experiments by placing mismatch repair under the control of an inducible promoter. We chose to translationally fuse mCherry to *mutH* so we could also study its affect on the mutation rate; *mutS* and *mutL* have been studied *in vivo* by (Elez *et al.* 2012). We quantify the likelihood of mismatch repair as a function of MutH concentration. We have done this by starting with strain ME121 (Elez *et al.* 2012), a strain of *E. coli* with *mutH* deleted and MutL under the control of an inducible *lac* promoter and fused to a yellow fluorescent protein. (Elez *et al.* 2012) used ME121 to quantify mutation rates in individual cells with high mutation rate by microscopy. Into ME121, we have placed a construct with expression of MutH under the control of the promoter $P_{LTetO1}$; this MutH construct was inserted into the chromosome to keep expression low. By tuning expression levels of MutH and MutL, we are able to vary the mutation rate of *E. coli* throughout a broad range; it is possible to vary the mutation rate over two orders of magnitude this way including intermediate points. This makes it possible to vary the mutation rate in evolution experiments and thus measure the effects of the mutation rate on evolutionary dynamics without varying other factors or constructing a new strain for each mutation rate to be tested.

## Materials And Methods

### Strains

All strains used in the experiment were varieties of *E. coli* K-12 MG1655. Some were constructed starting from MG1655 itself and others starting from the strain ME121 (Elez *et al.* 2012). The primary strains used in experiments throughout this paper were NS001 and NS001Δcat. These strains were engineered to have MutH expression controlled by a tetracyline induction system giving us fine control of the point mutation rate through mismatch repair. The only difference between them is that we removed chloramphenicol resistance from the Δcat strain.

**Figure 1:**
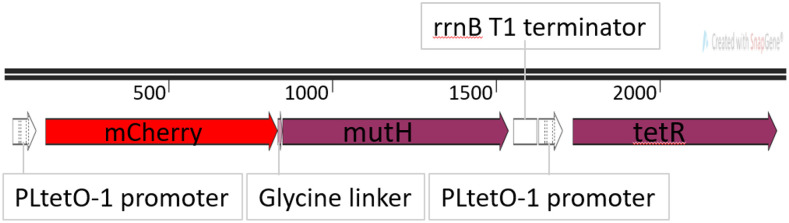
Diagram of the *mCherry-mutH* construct. *mCherry* is translationally fused to the N-terminus of *mutH*. Both *mCherry-mutH* and *tetR* are expressed from $P_{LTetO1}$ promoters, meaning their expression is normally suppressed but can be induced by anhydrotetracycline. The regulation of *tetR* expression by TetR protein makes the response of the system to anhydrotetracycline less sensitive (Nevozhay *et al.* 2009)

NS001 and NS001Δcat were constructed starting from ME121. A plasmid containing a construct of a translational fusion of mCherry to the N-terminus of MutH with a five glycine linker under the control of the promoter $P_{LTetO1}$ (Lutz and Bujard 1997) was synthesized *de novo* (GENEWIZ). The ribosomal binding site of this construct was the consensus Shine Dalgarno sequence for *E. coli*. A copy of the tet repressor gene was also placed under control of a $P_{LTetO1}$ promoter with a consensus ribosomal binding site. This construct was called pUC57(amp)-$P_{tet}$-mCherry-mutH-$P_{tet}$-tetR. A diagram of the construct without the pUC57(amp) backbone can be seen in Figure 1. Early experiments indicated the consensus ribosomal binding site upstream of *mCherry-mutH* expressed too much protein for our purposes, so the ribosomal binding site sequence was changed to be that of *lacI*. This was accomplished by performing PCR on the original construct with long primers that had the *lacI* ribosomal binding site in place of the consensus ribosomal binding site. This construct without the plasmid backbone was ligated into the CRIM plasmid pAH144 (Haldimann and Wanner 2001) using T4 DNA Ligase (NEB). pAH144-$P_{tet}$-mCherry-mutH(lacIRBS)-$P_{tet}$-tetR was then integrated into the chromosome of ME121 at the HK022 phage attachment site using the CRIM method (Haldimann and Wanner 2001). To further repress expression of mutH when uninduced, the medium copy number plasmid pTKIP-neo-$P_{tet}$-tetR where $P_{LTetO1}$ controls tet repressor expression was transformed into the strain after the *mCherry-MutH* construct was integrated into the chromosome. This strain including the plasmid, we call NS001. To make NS001 Δcat, before inserting pTKIP-neo-$P_{tet}$-tetR, we used pCP20 to flip out the cat gene using the method of (Datsenko and Wanner 2000).

MG1655 Δ*motA mCherry-mutH* is a modification of MG1655 with translational fusion of the protein mCherry to the N-terminus of MutH with a five glycine linker inbetween at the native *MutH* locus in the chromosome. It was constructed via the landing pad method (Tas *et al.* 2015). This strain was made to test that the protein MutH could still engage in mismatch repair after translational fusion to mCherry and to measure the expression level of the native *MutH* gene.

There are tables of the strains, plasmids, and PCR primers used in construction and verification in the Supplemental Experimental Procedures, strain construction.

### Mutation Rate Measurements

Mutation rates were measured using a rifampicin plating method (Rosche and Foster 2000). An overnight culture was diluted 1000-fold into fresh supplemented M9 (Elez *et al.* 2012) plus inducers and maintained in exponential growth for several doublings before being diluted to an OD of $10^{-7}$ in fresh medium plus inducers again. 250 μl aliquots of this low OD culture were then placed into a water bath shaker at 37${}^{\circ }$ C overnight. This overnight culture was allowed to grow for approximately 20 doublings until the cultures had reached an OD ∼ 0.1. The next day when this OD was reached, one μl from each aliquot was taken for a $10^{6}$ fold dilution into phosphate-buffered saline and 100 microliters of this dilution was plated on an LB plate. The remaining 249 μl of each aliquot was plated directly on to rifampicin plates (50 μl/ml). All plates were placed in a 37${}^{\circ }$ C incubator for 24 hr. Then they were removed and colonies were counted on each plate.

#### Inference of the mutation rate:

Mutation rates were inferred from plating data using the Ma-Sarkar-Sandri maximum likelihood method (Sarkar *et al.* 1992). Confidence intervals were determined using equations (24) and (25) in (Rosche and Foster 2000).

## Microscopy and Image Analysis

To measure the expression of mCherry-MutH, cells were grown in supplemented M9 (Elez *et al.* 2012) plus antibiotics overnight at 37${}^{\circ }$ C with 220 rpm shaking in a New Brunswick C76 water bath shaker. The next morning after saturation they were diluted 1000 fold into fresh supplemented medium and allowed to grow for five or six more doublings, then diluted again into fresh medium with the same antibiotics plus any needed inducers. If inducers were added, strains were allowed at least 5 more doublings before imaging. Cells were imaged in exponential phase at an optical density between 0.05 and 0.25. Serial dilutions were used to keep the optical density of all cultures below 0.25 at all times. The optical density at 600 nm ($OD_{600}$) of cultures was measured with a Bio-Rad SmartSpec Plus spectrophotometer.

When a culture was ready for imaging, a pad of M9 salts plus 1% agarose was prepared on a glass slide. Two 1 cm × 1 cm squares of agarose were cut out. On one square, 1 microliter of 10x concentrated Quantum QC # 3 beads (Bangs Laboratories, Inc.) was placed as a fluorescent reference standard. On the other square, 5 microliters of *E. coli* from a culture was placed. Then both squares were covered with a single no 1.5 glass coverslip and the slide was placed in a 37${}^{\circ }$ C incubator for 20 min to allow cells to settle on the pad. The slide was taken to the microscope where it was maintained at 37${}^{\circ }$ C by a temperature-controlled environmental chamber around the microscope. The microscope was a Nikon Eclipse Ti-E fully automated inverted microscope with Perfect Focus System automated focus correction. Cells were brought into focus in phase contrast and the perfect focus system activated. Then the microscope field of view was moved to the segment of agarose pad with fluorescent beads and the angles and focus of lasers were quickly adjusted to make sure illumination was bright and even. The stage was then moved back to the segment of agarose pad with *E. coli* and automated image acquisition was begun in a grid of 100-200 fields of view. After imaging the *E. coli*, we moved back to the agarose pad with the fluorescent beads and imaged beads in thirty-six fields of view. Images were taken taken using a Nikon CFI Apo TIRF 100x oil-immersion objective (N.A. 1.49). Fluorescent images were captured using an Andor iXon Ultra 897 EMCCD camera; phase contrast images were captured using a Nikon DS-Fi2 camera. Illumination for mCherry was provided by a Coherent Sapphire 561 nm laser and the exposure time of the Andor camera was 200 ms with 300 EM gain.

Image analysis was performed using custom python code. Intensities were adjusted for illumination differences using the reference beads and cell intensities have the local background subtracted. All intensities are given as a fraction of the reference beads intensity. We estimated the uncertainties in intensities by taking the mean difference between replicate experiments over the average of these experiments. This uncertainty was comparable to the uncertainty in the brightness of our reference beads when we put them on separate pads of agar next to each other so the uncertainty of our intensity measurements is likely dominated by the differences in illumination across a slide or our inference procedure. For a more detailed description of image analysis see the supplemental file Example_Microscopy_Analysis.html.

## Evolution Experiments

Our evolution experiment was done in a TECAN Infinite 200 platereader allowing us to measure the *E. coli* population in every condition and replicate every day. The strain was NS001Δcat. The culture medium used was M9 minimal medium supplemented with 0.2% w/v casamino acids. Antibiotics spectinomycin (100 μg/ml) and ampicillin (100 μg/ml) as well as plus varying amounts of the inducers that control the point mutation rate in this strain were added as needed. Cultures were grown in a 48-Well CytoOne plate, nontreated. The temperature of growth was 30${}^{\circ }$ C, and the plate was shaken in orbital mode with a frequency of 280.8 rpm and an amplitude of 2 mm. A temperature of 30${}^{\circ }$ C was chosen because it is colder than the optimal temperature of growth for *E. coli* 37${}^{\circ }$ C; we chose a suboptimal environment for growth because we wanted to see how the process of evolutionary adaptation to a suboptimal environment depended on the mutation rate. Every 10 min, the optical density at 600 nm was measured by the TECAN. Each plate was arranged to have nine replicates of five mutation rate conditions and three blanks. We call these mutation rate conditions Low, LoMid, Mid, HiMid, and High. The mutation rates of each of these conditions and the inducer concentrations necessary to reach these mutation rates are shown in Table 1. IPTG controls *mutL* expression, and anhydrotetracyline (aTc) controls *mutH* expression. Cells grew in the platereader for the entire day except when transferring cells between plates. Each day, the wells of a fresh plate were filled with 500 *μ*l of fresh medium and 1 *μ*l of cells from the matching well of the day before. Plating experiments with the same medium, temperature, and platereader settings indicated that the initial number of colony-forming units that started in each well each day after transfer was on the order of one to ten million (data in ”Estimating NS001 per OD600.html”). Samples from all wells were frozen every 3 days to allow for resuming the experiment and other purposes. For the layout of the conditions and replicates in a plate and further details on transferring cells from day to day, see Supplemental Information Figure 1. For a description of growth curve analysis, see the supplemental file Notebook_Evolution_Experiments.html.

**Table 1:** Mutation rate conditions for evolution experiment

Condition Name	Mutation Rate	95% confidence interval	[IPTG] (μM)	[aTc] (ng/ml)
High	$2.2\times 10^{-7}$	$\left(1.6,2.9\right)\times 10^{-7}$	0	0
HiMid	$4.1\times 10^{-8}$	$\left(2.6,5.9\right)\times 10^{-8}$	50	0
Mid	$1.4\times 10^{-8}$	$\left(.64,2.5\right)\times 10^{-8}$	2000	0
LoMid	$3.8\times 10^{-9}$	$\left(1.2,7.4\right)\times 10^{-9}$	2000	2
Low	$1.7\times 10^{-9}$	$\left(.41,3.6\right)\times 10^{-9}$	2000	10

## Genome Sequencing

After the evolution experiment finished, twenty wells at two timepoints each were chosen for sequencing. The ancestral strain NS001Δcat and its ancestor ME121 were also sequenced. For the evolved strains, samples of the first four replicates from each mutation rate condition (Table 1) were sequenced from day 24 and day 41 (the final day).

For sequencing, all samples were taken from frozen stocks and grown overnight in 2 mL of LB with 2 mM IPTG and 10 ng/ml aTc. One sample was grown for each evolved replicate at days 24 and 41, and one sample of ME121 was grown. Three samples of the ancestor NS001Δcat were grown to improve coverage and serve as a test of sequencing and analysis replicability.

Genomic DNA was extracted from the samples using the DNEasy UltraClean Microbial Kit. Following DNA extraction, a library was prepared for sequencing using the NEXTERA XT library prep kit with Nextera XT v2 Index kit A used for the indices of the 44 samples. DNA concentrations were measured using a Clariostar Plus Microplate Reader. After library clean up and before normalizing, the DNA was analyzed using an AATI Fragment Analyzer at the Roy J. Carver Biotechnology Center to measure DNA fragment length and concentration. Normalization was performed by following the bead based normalization steps. Pooled libraries were sequenced with the MiSeq Reagent Kit v3 (600 cycle) on an Illumina MiSeq in the Center for the Physics of Living Cells. Data output was FASTQ only.

Data were analyzed using the program breseq; breseq outputs a list of probable mutations of various types, the sequence evidence for them, and a statistical estimate of mutation frequency in a sample (”Deatherage and Barrick ”2014”). All analyses were run in polymorphism mode. First the .fastq files of all three samples of the ancestor were run against the reference sequence NC_000913.3 for E.coli MG1655 from NCBI with the annotations for the multiple copies of the *yahH* gene numbered. The remaining samples used an annotated sequence of the the genome of NS001 as a reference. This annotated sequence was made from mutations found by breseq as compared to the NCBI MG1655 sequence by using the APPLY command of the gdtools utility in breseq. Each individual sample of the ancestor NS001 was also compared to the aggregated samples of NS001, and breseq was run over the aggregate samples again comparing them to their own reference in order to separate polymorphisms in the ancestral population NS001 from fixed mutations.

breseq estimated the coverage of all samples was 30x or greater; all but two samples had coverage of 50x or greater. Across all samples, the mean fraction of sequencing reads mapped to the reference was 94% with a minimum of 90% and a maximum of 95%. We considered the coverage, reads mapped, and quality to be high enough to include all samples in further analysis.

We used the mutation data from breseq to estimate the cumulative number of single nucleotide polymorphisms (SNPs) as a function of polymorphism frequency starting from high frequency (fixation) going to low frequency. We did not count any mutations found in the ancestor at any frequency for calculations of the cumulative curve in order to focus on mutations that likely occurred *de novo*. We also did not count any mutations found in more than one well since we believe these were mutations that were present in the ancestor at low frequency. We cannot guarantee we removed rare mutations (<5%) present in the ancestor from consideration. We calculated the means of these cumulative curves across all samples in a given mutation rate condition to get a smoother estimate of how the the number of SNPs varies with the mutation rate.

We used the mutation data from breseq to look for signs of selection in particular genes. We considered each type of mutation found separately: substitutions, deletions, insertions, and mobile element mutations. To increase our statistical power and because all strains evolved in the same environment, we aggregated the mutations found across all evolved samples but not in the ancestor for this analysis. Once again, we also did not count any mutations that were found in more than one well since we believe these were rare mutations present in the ancestral population. To be conservative, we calculated the probability of finding the number of unique mutations found in a given gene to the chance of finding any cluster of mutations in a segment of the genome of the same length given the total number of mutations found by breseq and the length of the genome under the assumption that mutations were equally likely to occur anywhere in the genome. This probability was calculated by simulation. This comparison corrects for the multiple comparisons problem of searching the entire genome for genes with multiple mutations.

### Data availability

Strains and plasmids are available upon request. Files SNPs.html, Polymorphisms.html, Microscopy-Analysis.html, and Evolution-Experiments.html contain code and detailed descriptions of experimental procedures and analyses. Sequence data are available at the NCBI Sequence Read Archive with accession number PRJNA589707. Supplemental material available at figshare: https://doi.org/10.25387/g3.12437177.

## Results

### Mismatch repair continues to function After translationally fusing mCherry to mutH

Rifampicin plating tests confirmed that the mutation rate is unchanged from wildtype after fusing mCherry to the N-terminus of the native copy of *mutH* in the chromosome. We compared MG1655 to MG1655 Δ*motA mCherry-mutH* and ME120 to NS001 at close to wildtype levels of MutH using a 2-sample Kolmogorov-Smirnov test and found no significant difference in mutation rate (Table 2)

**Table 2:** Effect of translationally fusing mCherry to MutH on the mutation rate

base strain	MG1655	ME120
mutation rate	$10\times 10^{-9}$	$3\times 10^{-9}$
95% confidence interval	$\left(5,18\right)\times 10^{-9}$	$\left(.9,6\right)\times 10^{-9}$
strain after mCherry-MutH fusion	MG1655-*mCherry-mutH*	NS001
mutation rate	$8\times 10^{-9}$	$2.0\times 10^{-9}$
95% confidence interval	$\left(3.5,14\right)\times 10^{-9}$	$\left(.8,4\right)\times 10^{-9}$
p-value of 2-sample Kolmogorov-Smirnov test	0.70	0.72
mutation rate affected by mCherry-MutH translational fusion	No	No

### MutH expression can be varied by two orders of magnitude

**Figure 2:**
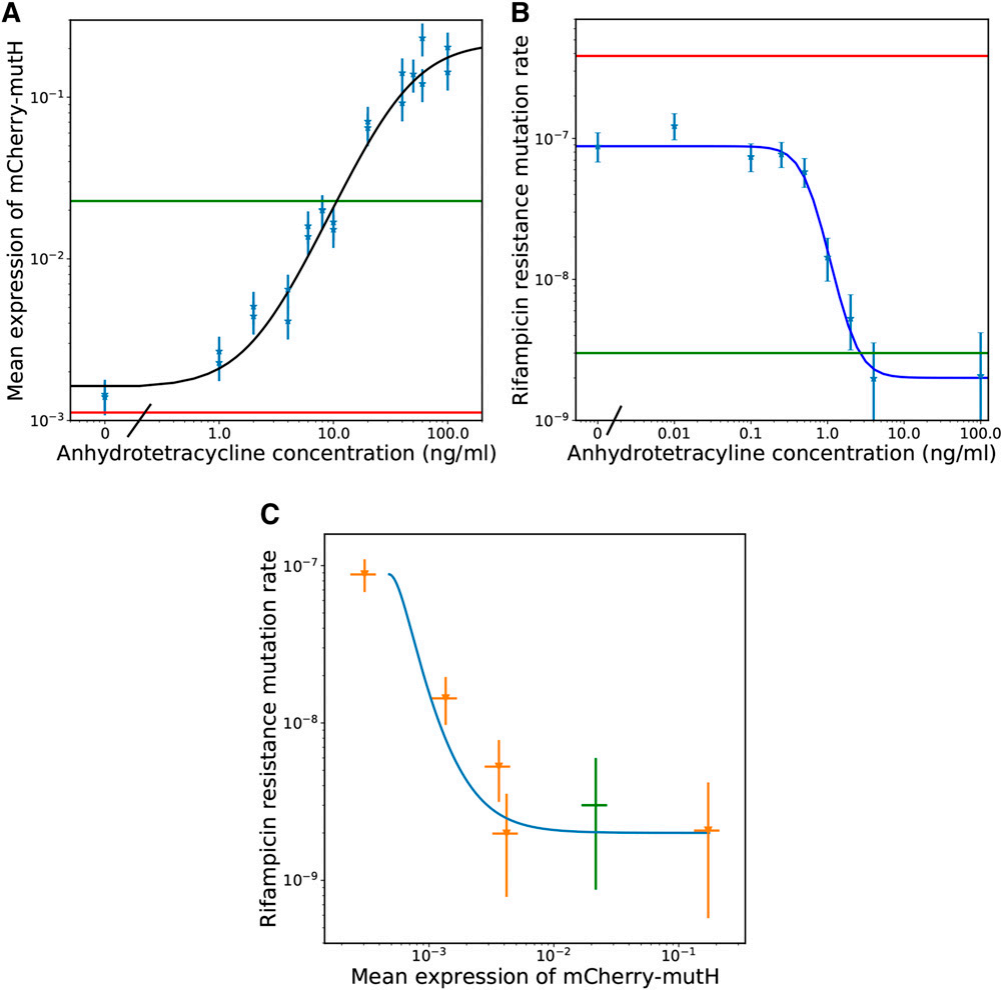
a Mean mCherry-MutH expression as a function of the concentration of the inducer of mCherry-MutH expression anhydrotetracycline (aTc). The green line is the expression of mCherry-MutH from the wildtype locus which is not sensitive to anhydrotetracycline (aTc). The red line is the level of fluorescence measured from a control strain (ME121) which expresses no mCherry. The light blue points are the mean expression measured by fluorescent imaging. The dark blue curve is a Hill function plus shift fit to that data $y=\frac{A}{\left(1+{\left(\frac{k_{a}}{x}\right)}^{n}\right)}+C$. The parameters were A=.22, $k_{a}=42$, n=1.6, and $C=1.6\times 10^{-3}.$ b The mutation rate to rifampicin resistance per cell division as a function of the aTc concentration. The red line is the mutation rate of ME121 which is defective for mismatch repair. The green line is the mutation rate of ME120 which has fully functioning mismatch repair. The light blue points are data from NS001 at different levels of aTc induction of *mutH* expression. *mutL* induction was saturated with 2000 *μ*M of IPTG. The dark blue curve is a Hill function plus shift fit to that data $y=\frac{A}{\left(1+{\left(\frac{k_{a}}{x}\right)}^{n}\right)}+C$. The parameters were $A=-8.6\times 10^{-8}$, $k_{a}=.57$, n=2.9, and $C=8.8\times 10^{-8}.$ c The mutation rate to rifampicin resistance per cell division *vs.* the expression of mCherry-MutH. The orange data are MutH expression and mutation rates of NS001 grown with varying concentrations of aTc. The green data are the expression of MutH from the wildtype locus and the mutation rate of ME120. The blue curve uses the mean expression of mCherry-MutH from the hill curve fit of expression *vs.* aTc as x and the mutation rate from the hill curve fit of mutation rate *vs.* aTc as y.

MutH expression levels can be varied from approximately 10 fold below the level of wildtype expression to 10 fold above the level of wildtype expression; the expression as a function of inducer concentration is well described by a hill function (Figure 2a). The response is not sensitive, with a hill coefficient of 1.6.

When the aTc concentration is 1 ng/ml or less, the mean expression is only distinguishable from zero by averaging over the intensity of hundreds of cells. The standard deviation of the autofluorescent intensity of the Δ*mutH* ME121 strain which expresses no MutH is slightly greater than the mean expression at 1 ng/ml of aTc. Past this level of induction, however, fluorescence in individual cells becomes apparent by eye.

### The expression of MutH can be adjusted to vary the point mutation rate by two orders of magnitude

By adjusting the amount of MutH expressed, we can vary the point mutation rate from a factor of 3 lower than a complete MutH knockout to the level of a wildtype. This is a range of point mutation rates over two orders of magnitude. Across replicate experiments, we are able to control/measure the point mutation rates in this range to a factor of approximately 3. The full curve of anhydrotetracycline concentration *vs.* mutation rate can be seen in Figure 2b. That no induction still doesn’t quite reach the mutation rate of a *mutH* knockout strain shows the *mCherry-mutH* construct is still slightly leaky in expression. However, since NS001 descends from ME121 which expresses *mutL* from the *lac* operon in the chromosome, we found we can affect the mutation rate through the inducer IPTG as well, allowing us to reach the mutation rate of a ΔmutH strain. However, the response curve to IPTG is highly sensitive (Supplemental Information - Mutation rate as a function of MutL induction).

### Expressing more MutH than the native locus does not lower the mutation rate

The mutation rate of ME120 was $3\times 10^{-9}$ with a 95% confidence interval of $\left(.9\times 10^{-9},\;6\times 10^{-9}\right)$. The mutation rate of NS001 with 2000 *μ*M IPTG and 100 ng/ml aTc was $2.1\times 10^{-9}$ with a 95% confidence interval of $\left(.6\times 10^{-9},\;4.2\times 10^{-9}\right)$ . These mutation rates are close together and have largely overlapping confidence intervals indicating little change in mutation rate, even though at this level of induction, the mean expression of MutH in NS001 exceeds that of the wildtype by a factor of almost 10.

## Evolved strains of *E. coli* at varying mutation rates

**Figure 3:**
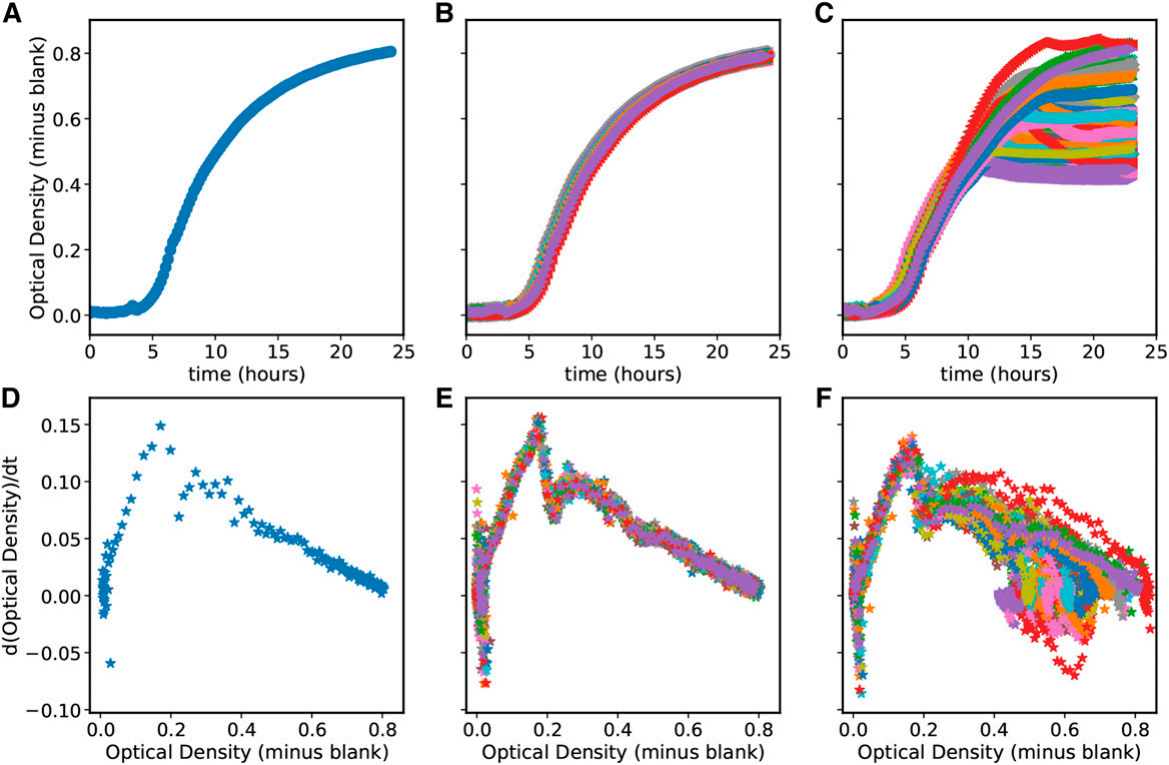
First row - optical density over time for (A) A single well on the first day of the experiment, (B) All wells on the first day, and (C) All wells on the last day of the experiment. Second row - first derivative of optical density over time plotted *vs.* the optical density for (D) the same well as in A, (E) the same wells as in B, and (F) the same wells as in C.

Since the evolution experiment was performed in a platereader, we have curves of the optical density over time for every replicate on every day. As an example, the optical density over time for the first replicate of the high mutation rate wells on the first day is plotted in in Figure 3A, and the derivative of optical density over time *vs.* the optical density of the same well is shown in Figure 3D. Initially, all wells had roughly the same growth curve, as seen in Figures 3B and E. In the graphs of the derivative of optical density *vs.* optical density, there are two linear regions of the growth dynamics and a transition in between. Up to an optical density of 0.16, optical density grows exponentially. However, at very low optical densities, the platereader is not sensitive enough to get good measurements of growth, as shown by the scatter of points at very low OD in the graphs of the derivative of optical density. At optical densities above 0.3, optical density decays toward a maximum at a different exponential rate. This region is less linear for some replicates later in the experiment. Over the more than 1000 optical density curves measured, we found that all but five curves had the same behavior at low optical densities. Such curves typically do not repeat themselves and show erratic behavior that we believe is likely to be a transient error from the platereader rather than a change in the growth of *E. coli*. As shown in Figure 3F, for all wells the early exponential phase of growth on the final day of the experiment is approximately the same as it was on the first day.However, we can see in Figure 3F that the behavior of the growth curves at higher optical densities has drastically changed over the course of the experiment and has diverged from well to well. Many wells have lower final optical densities by the end of the experiment. We will elaborate on these changes below.

### The induced mutation rates are stable on the timescale of the experiment

For this strain to be useful for investigating the effect of the mutation rate on evolution, it must have a mutation rate that is reasonably stable over at least hundreds of generations. We checked that this was the case by performing rifampicin plating tests on seven of the evolved replicates in the platereader. For four of the seven wells tested, the inferred mutation rates were within the 95% confidence intervals of the ancestral strain mutation rate measurement. The two conditions at High and HiMid mutation rates were just outside the 95% confidence interval of the ancestral strain mutation rate measurement but within a factor of 2 of the original mutation rates. This is comparable to when we repeat measurements with the ancestor strain at high mutation rates. Consequently, we believe our confidence intervals underestimate the true experimental error. Finally, over the course of the experiment one well spontaneously developed rifampicin resistance. The data on the mutation rates and confidence intervals for these evolved strains can be found in Table S4.

### Strains evolved in a platereader show changes in the saturating and possibly lag phases of growth but not in exponential phase

**Figure 4:**
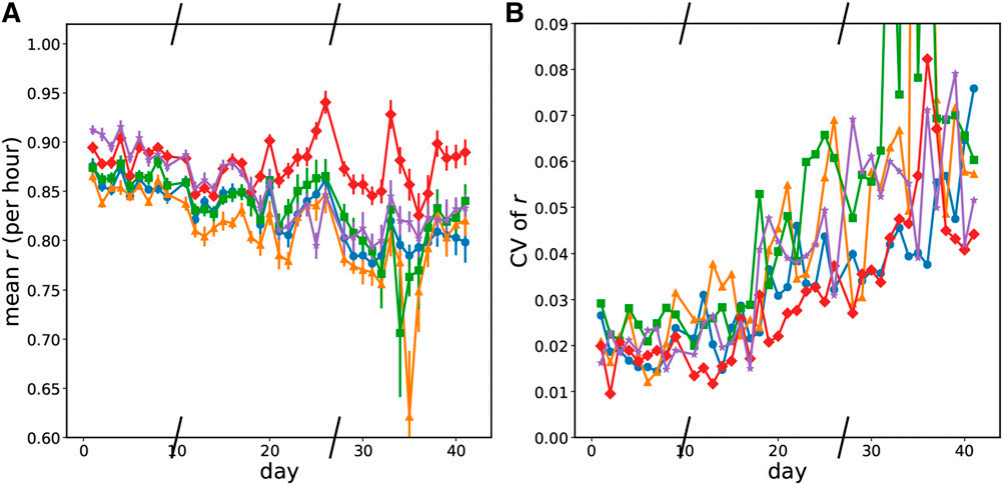
Evolution of the growth rate in early exponential over time. Slashes have been placed on the x-axis where the experiment was frozen and resumed later. Blue circles - Low mutation rate; orange triangles - LoMid mutation rate; green squares - Mid mutation rate; red diamonds - HiMid mutation rate; purple stars - High mutation rate. a Mean growth rate in exponential phase by mutation rate condition over time. b Coefficient of variation of the the growth rate in exponential phase by mutation rate condition over time.

**Figure 5:**
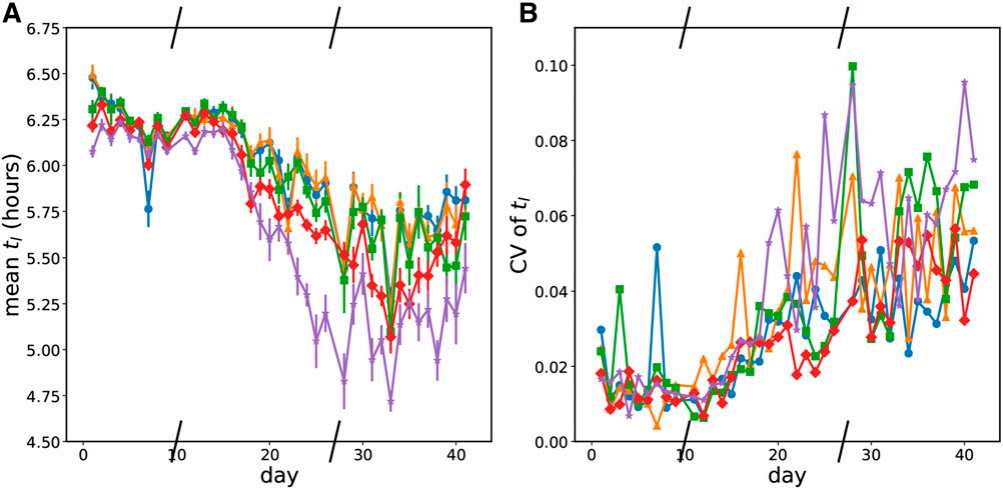
Evolution of the time before leaving exponential phase growth a The mean time before the optical density exceeded that of exponential phase growth (OD600 = 0.16) by mutation rate condition. Blue circles - Low mutation rate; orange triangles - LoMid mutation rate; green squares - Mid mutation rate; red diamonds - HiMid mutation rate; purple stars - High mutation rate. b The coefficient of variation of the time before the optical desnity exceeded that of exponential phase growth by mutation rate condition. Blue circles - Low mutation rate; orange triangles - LoMid mutation rate; green squares - Mid mutation rate; red diamonds - HiMid mutation rate; purple stars - High mutation rate.

Over the 42 days of the platereader experiments, we did not observe large changes in *E. coli* growth rate in exponential phase from $∼.85\;{\text{hr}}^{-1}$ in any conditions for any replicates (Figure 4). Some replicates showed no change in exponential growth rate while some showed small decreases in the exponential growth phase. However, after roughly 15 days, there began to be clear changes in the saturating optical density at the end of the day and in the growth approaching that optical density. The cultures also reached the optical density that separates exponential phase growth from nonexponential phase growth (OD600 0.16) earlier. We call the time to reach an OD600 of 0.16 ”time to leave exponential phase” because of the shape of the derivative of optical density as a function of optical density (Figures 3D-F). So even though the exponential phase growth rate was unchanged, either the lag phase shortened or more cells existed at the end of saturation phase when the daily transfer occurred. The length of lag phase is connected to the conditions of the stationary culture before transfer (Baranyi and Roberts 1994; Jõers and Tenson 2016). Because we use a supplemented minimal medium, our cells likely only go through a ”lag 2” phase of growth without cell division (Madar *et al.* 2013). Unfortunately, at the initial low optical densities of inoculation, we are unable to get a good measure of this period from our growth curves but can conclude that it is less than three hours. Hence, we cannot use growth curves to distinguish a rise in the initial number of cell inoculum from a change in lag phase. The time to leave exponential phase was a noisy measurement, but all wells show a decrease over the course of the experiment. Higher mutation rates show a larger and faster decrease; this can be seen in the mean value at a given mutation rate of the time to leave exponential (Figure 5a). Given that the exponential growth rate of all wells is 0.85, the roughly 1 hr decrease in time to leave exponential in the High mutation rate condition corresponds to a little over an additional doubling of the population. In the Low and LoMid conditions, the time to leave exponential decreases by only half that which corresponds to 1.5 times more population. Thus on average, fitness at transfer and/or in lag phase increased increasingly quickly at high mutation rates.

The shortened time to leave exponential phase does not correspond to increases in the saturating optical density of each well. We observed wells where saturating optical density increased or decreased over time for several days while time to leave exponential phase decreased. Because changes in both directions occurred from the initial growth curves, we interpret the changes in saturating growth as being correlated with whatever traits were being selected for rather than being the target of selection itself. Most replicates initially showed an increase in saturating optical density followed by a decrease, however some only showed a decrease.

### Declines in saturating optical density happen sooner at the highest mutation rate

**Figure 6:**
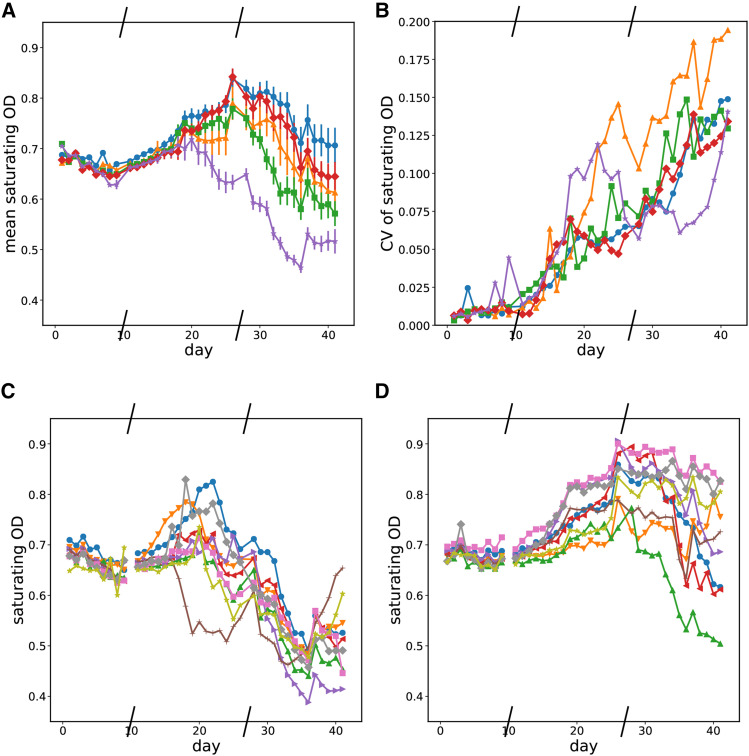
Evolution of saturating optical density over time. Slashes have been placed on the x-axis where the experiment was frozen and resumed later. a - mean saturating optical density by mutation rate condition over time. Blue circles - Low mutation rate; orange triangles - LoMid mutation rate; green squares - Mid mutation rate; red diamonds - HiMid mutation rate; purple stars - High mutation rate. b - Coefficients of variation of saturating optical density by mutation rate condition over time. Blue circles - Low mutation rate; orange triangles - LoMid mutation rate; green squares - Mid mutation rate; red diamonds - HiMid mutation rate; purple stars - High mutation rate. c - Saturating optical density over time for replicates in the high mutation rate condition. d - Saturating optical density over time for replicates in the low mutation rate condition.

Halfway through the experiment, we start seeing significant declines in saturating optical density in replicates in multiple mutation rate conditions. However, this occurs the soonest and by far the most consistently in our highest mutation rate condition. This is seen in the mean behavior of the saturating optical density across all replicates at a given mutation rate in Figure 6a or by comparing the changes in saturating optical density in individual wells in the High 6c and Low 6d mutation rate conditions. Graphs of the saturating optical density each day for the other conditions are in the supplemental file Notebook_Evolution_Experiments.html.

### Saturating optical density and time to leave exponential diverge Over time Between replicates at the same mutation rate

Evolution is a stochastic process, and we find that even in the same condition, growth does not always evolve the same way. We find that the divergence between replicates increased over the course of our experiment for growth rate, time to leave exponential, and saturating optical density. We quantified the divergence between replicates at a mutation rate by the coefficient of variation of each growth parameter on each day. Examining the data in Figures 4b, 5b, and 6b, there is an overall trend of an increase in all coefficients of variation over time, but we do not observe obvious trends in how quickly growth curve parameters diverge from each other with respect to the mutation rate condition.

## Genome Sequencing Results

**Figure 7:**
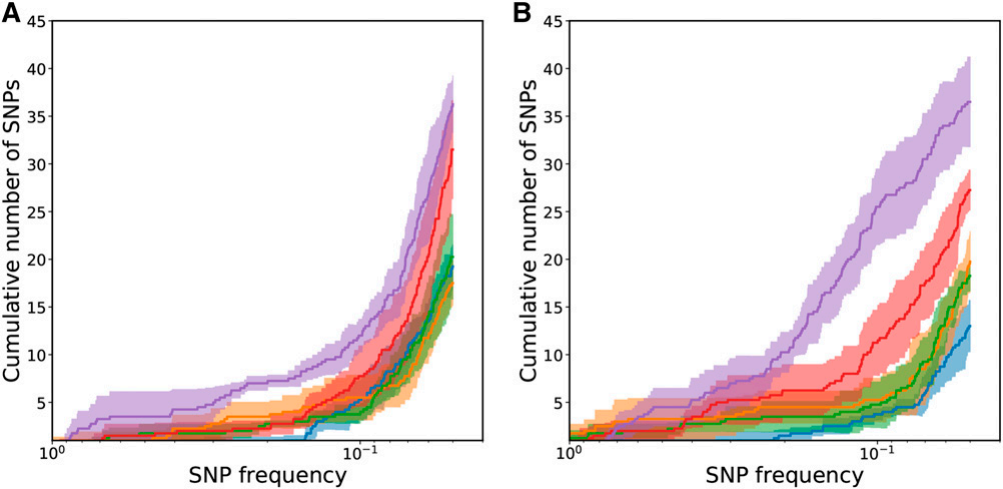
The mean number of cumulative SNPs for each mutation rate condition. Shading is an estimate of the standard error of the mean for each mutation rate condition. Blue - Low mutation rate; orange - LoMid mutation rate; green - Mid mutation rate; red - HiMid mutation rate; purple - High mutation rate. a is on day 24. b is on day 41.

The mean cumulative number of SNPs starting with fixed mutations and moving toward rare mutations is plotted for each day and each mutation rate condition in Figure 7. The increasing slope of the cumulative graph indicates low frequency single nucleotide polymorphisms (SNPs) were more common than higher frequency SNPs or fixed mutations. Although the High mutation rate condition has the largest number of SNPs and the HiMid mutation rate condition the second largest number of SNPs, the mean cumulative number of SNPs for the remaining mutation rate conditions are fairly tightly clustered. Additionally, the fold change in the total number of SNPs between High and Low mutation rate conditions is ≈3 even though the mutation rate fold change is ≈130. This can be explained by the action of selection during the course of evolution. Due to our ≈50x coverage, we use breseqs normal cutoff for polymorphisms calls and do not call mutations below a frequency of 5%. Since the total population size in a well at the end of day is on the order of a billion and the transferred population to the next well is on the order of a million, only beneficial mutations or mutations linked to them can reach a frequency as high as even 1%, much less 5%.

Over all genes, we found 825 substitution mutations, 35 deletions, 18 insertions, and 3 mobile element mutations. We only found one gene which had a cluster of substitution mutations that was statistically significant on the whole genome level. *sufB* had 8 distinct mutations and is 1488 basepairs long. This corresponds to a p-value of $\approx 8\times 10^{-5}$ under the null hypothesis of 1000 substitution mutations uniformly and randomly distributed in the chromosome. We found only one gene which had a cluster of deletion mutations that was statistically significant on the whole genome level as well. *sufS* had 3 distinct deletion mutations and is 1221 basepairs long. This corresponds to a p-value of 0.002 under the null hypothesis of 40 deletions uniformly and randomly distributed throughout the chromosome. No gene had more than one insertion mutation or mobile element mutation across all samples and so no clustering was found in these. Notably, 1 of the 18 insertion mutations and 1 of the 3 mobile element mutations were in *sufS*. Since *E. coli* have thousands of genes, and we found so few of these types of mutations, it is suggestive that in addition to the statistically significant cluster of deletions in *sufS* there was also an insertion and a mobile element mutation, suggesting that there is selective action deactivating *suf* gene function.

Because we found a statistically significant cluster of substitution mutations in *sufB*, many rarer types of mutations in *sufS*, and a not statistically significant cluster of 3 mutations in *sufC*, we also checked the significance of the total number of substitution mutations found across all evolved strains in the *sufABCDSE* operon. The total length of the operon is 5513, and across all sequenced samples there were 0 mutations in *sufA*, 8 in *sufB*, 3 found *sufC*, 0 in *sufD*, 1 in *sufS*, and 1 in *sufE* for a total of 13 mutations. Two of these thirteen mutations were nonsense mutations, which adds another small amount of evidence for selective action deactivating *suf* gene function. This number of mutations in this operon corresponds to a p-value of $\approx 3\times 10^{-6}$ which is significant and smaller than the p-value considering *sufB* alone. However, differences in statistical significance are not necessarily statistically significant so we make no claim that the operon is necessarily more sensibly considered the region of enhanced mutation than some of the individual genes on their own.

## Discussion

By placing *mutH* under the control of an anhydrotetracyline inducible system and translationally fusing it to *mCherry*, we have measured the dependence of the mutation rate on the concentration of MutH protein and made a strain of *E. coli* where it is possible to continuously vary the point mutation rate across two orders of magnitude.

We found that over the course of an evolution experiment to adapt to growth at 30${}^{\circ }$ C, changes in growth occurred in all replicates in saturating optical density and time to leave exponential phase. Time to leave exponential phase decreased most quickly on average in the High then HiMid mutation rate conditions. That changes in time to leave exponential phase were adaptive can be seen by how replicates reached an optical density of 0.16 more quickly at the end of the experiment than at the beginning. Saturating optical densities increased over time at first in most replicates then decreased. However, in the High mutation rate condition, many replicates had a shortened or nonexistent period of increase in saturating optical density.

From sequencing, we found strong signs of selection in the *sufABCDSE* operon. SufS, together with SufE, acts as a cysteine desulfurase in oxidative stress conditions (Dai and Outten 2012). That most of the mutations in *sufS* were insertions, deletions, or mobile element mutations suggests that the regular functioning of SufS was deleterious in the environment in which we evolved the bacteria, although its exact role remains unclear. We also found that more polymorphisms occurred at higher mutation rates (threefold more mutation occurred in the high mutation rate condition compared to the low mutation rate condition), but the number of polymorphisms did not increase nearly as many fold as the difference in mutation rate between the high and low mutation rate condition (130-fold). The number of polymorphisms depends upon both the mutation rate in a population and the distribution of fitness effects due to mutations. Our strain and data open up the possibility of experimentally testing models of how the mutation rate affects the dynamics of the spread of beneficial mutations.

Being able to control mutation rates is important to experimentally investigating evolutionary dynamics. This has important practical applications because hypermutators are often found in antibiotic resistant clinical isolates of pathogenic bacteria (Denamur *et al.* 2000; Komp Lindgren *et al.* 2003). (Giraud *et al.* 2001) found mutators had an advantage in colonizing mouse guts in an in-vivo experiment; antibiotics killing bacteria in an environment may leave new niches for colonization which may explain part of the advantage hypermutators have in environments with antibiotics. Long term chemostat experiments with a continually rising antibiotic concentration were used to study continuing adaptation to antibiotics by (Toprak *et al.* 2011). It would be interesting to see how these dynamics are affected by changing the mutation rate. Mutators may be selected for in novel or rapidly changing environments because a larger number of beneficial mutations will usually be available in an environment an organism is less well adapted to than one it is already well adapted to. Novel environments change the balance in linkage between mutator phenotypes and beneficial mutations *vs.* linkage between mutator phenotypes and deleterious mutations. This changes the selection pressure on the mutation rate because selection on the mutation rate depends on the distribution of fitness effects due to mutations (Good and Desai 2016). However, as a population adapts to a new environment the frequency of beneficial mutations will likely drop and the linkage between mutator phenotypes and deleterious mutations found in the genome even during successful adaptation of mutators (Couce *et al.* 2017) will push mutation rates back down in the longer term once there are not frequent enough beneficial mutations to balance out the accumulation of deleterious mutations. This could be quantified better by testing a range of mutation rates in experimental environments. (Luan *et al.* 2013b) found that different environments favored different strengths of mutator *E. coli* when evolving mixtures of *E. coli* with plasmids containing various mutations to *dnaQ* to develop *E. coli* with tolerance to various toxic chemicals.

Being able to adjust the mutation rate more easily should make it easier to test theoretical models of adaptation in large populations like bacteria (Neher and Hallatschek 2013; Good and Desai 2016). Adaptation in these models also depends on the distribution of fitness effects, and in particular the set of fixed beneficial mutations depends on the mutation rate (Good *et al.* 2012). The distribution of fitness effects has been measured in mutation accumulation experiments, by sequencing, and recently by tracking single lineages after a mutation (Robert *et al.* 2018). However, beneficial mutations are very rare and any inference on the size and frequency of their effects using sequencing of adapting populations will need to account for the effects of the mutation rate on their fixation described in (Good *et al.* 2012). Tests of both the model and inferences about the distribution of beneficial fitness effects could be stronger if the mutation rate in evolution experiments is varied in a controlled way.

The range of mutation rates that could be reached by tuning could also be extended by expressing mutant polymerase proofreading subunits in the cell. (Chou and Keasling 2013) varied expression of the mutD5 mutant dnaQ polymerase subunit in response to metabolite production in order to dynamically adjust the mutation rate downward as *E. coli* evolved and began producing a desired metabolite. The errors made by the *mutD5* mutation can be partially compensated for by overexpression of mismatch repair proteins mutL and mutH (Schaaper and Radman 1989). So a system where expression of both a defective proofreading subunit and mismatch repair is adjustable would give control over two multiplicative factors feeding into the overall mutation rate. This may extend the adjustable range of mutation rates by a factor of 10 or 100 upwards since some *dnaQ* mutants have a mutation rate almost 10,000 times higher than wildtype (Taft-Benz and Schaaper 1998) whereas our strain can only reach mutation rates about 100 times higher than wildtype. It may be possible that overexpression of multiple mismatch repair proteins is able to drive the mutation rate below wildtype levels even though overexpressing MutH did not. So by placing mutS and/or mutL under the control of inducible promoters as well it may be possible to extend the range of tunable mutation rates downward; (Foster 1999) found that overexpression of mutS, mutL, or both reduced the number of reversion mutations of the*lac* or tet alleles. (Luan *et al.* 2013a) adjusted the mutation rate of *Clostridium acetobutylicum* by altering mutS and mutL levels, and did not attain lower than wildtype mutation rates.

We were able to quantify the effect of MutH numbers on the mutation rate by fusing it to mCherry fluorescent protein. MutS and MutL can also function when fused to fluorescent proteins, and (Elez *et al.* 2012) did this to study their stoichiometry at mismatch repair sites. Mismatch repair proteins are in a race with DAM methylation proteins and overexpression of DAM methylation can disable mismatch repair (Lennen *et al.* 2016). By tagging the Dam methylase and putting it under the control of an inducible promoter, the interactions of the mutSLH proteins and their race with the methylation system could be observed and the point mutation rate’s dependence on all four proteins quantified.
